# Supplementary material for: Tead1a Initiates Transcriptional Priming Through the TEAD1a/YAP‐Notch1‐Spi1/Cebpα Axis to Promote Neutrophil Fate
Source: Adv Sci (Weinh). 2025 Aug 28;12(41):e05441. doi: 10.1002/advs.202505441 (PMC12591127; doi:10.1002/advs.202505441)
Supplement: Supplementary file 4 — Supplementary Dataset [file ADVS-12-e05441-s004.zip › 3-4.zip!/3-4/02-4.cluster/cluster9/Enrich/ego_MF.pdf]

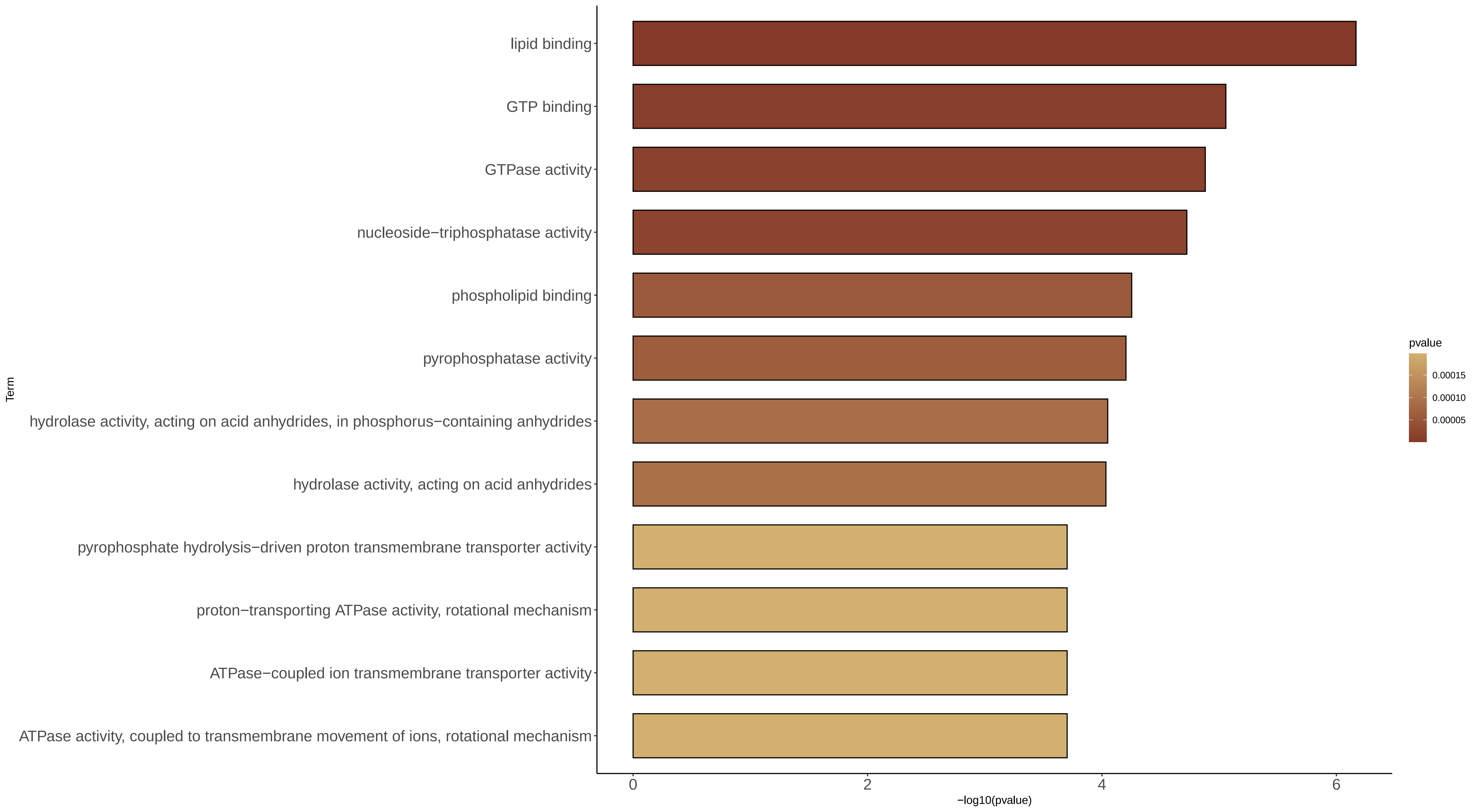
lipid binding
GTP binding
GTPase activity
nucleoside−triphosphatase activity
phospholipid binding
pyrophosphatase activity
hydrolase activity, acting on acid anhydrides, in phosphorus−containing anhydrides
hydrolase activity, acting on acid anhydrides
pyrophosphate hydrolysis−driven proton transmembrane transporter activity
proton−transporting ATPase activity, rotational mechanism
ATPase−coupled ion transmembrane transporter activity
ATPase activity, coupled to transmembrane movement of ions, rotational mechanism
Term
0
2
4
6
−log10(pvalue)
pvalue
0.00015
0.00010
0.00005
